# Enhanced Prediction of Hot Spots at Protein-Protein Interfaces Using Extreme Gradient Boosting

**DOI:** 10.1038/s41598-018-32511-1

**Published:** 2018-09-24

**Authors:** Hao Wang, Chuyao Liu, Lei Deng

**Affiliations:** School of Software, Central South University, Changsha, 410075 China

## Abstract

Identification of hot spots, a small portion of protein-protein interface residues that contribute the majority of the binding free energy, can provide crucial information for understanding the function of proteins and studying their interactions. Based on our previous method (PredHS), we propose a new computational approach, PredHS2, that can further improve the accuracy of predicting hot spots at protein-protein interfaces. Firstly we build a new training dataset of 313 alanine-mutated interface residues extracted from 34 protein complexes. Then we generate a wide variety of 600 sequence, structure, exposure and energy features, together with Euclidean and Voronoi neighborhood properties. To remove redundant and irrelevant information, we select a set of 26 optimal features utilizing a two-step feature selection method, which consist of a minimum Redundancy Maximum Relevance (mRMR) procedure and a sequential forward selection process. Based on the selected 26 features, we use Extreme Gradient Boosting (XGBoost) to build our prediction model. Performance of our PredHS2 approach outperforms other machine learning algorithms and other state-of-the-art hot spot prediction methods on the training dataset and the independent test set (BID) respectively. Several novel features, such as solvent exposure characteristics, second structure features and disorder scores, are found to be more effective in discriminating hot spots. Moreover, the update of the training dataset and the new feature selection and classification algorithms play a vital role in improving the prediction quality.

## Introduction

Proteins and their interactions play a pivotal role in most complex biological processes, such as cell cycle control, protein folding and signal transduction. The study of Protein-Protein Interactions (PPIs) is significant for the understanding of the complex mechanisms in a cell^1,2^. More importantly, protein-protein interactions are usually integrated into biological interaction networks for their interdependence, so that any erroneous or disrupted PPIs can cause disease. Studies of principles governing PPIs have found that energies are not homogeneous in protein interfaces. Instead, only a small portion of interface residues called hot spots contribute the majority of the binding energy^3^. Identifying these hot spot residues within PPIs can help us better understand PPIs and may also help us to regulate protein-protein binding.

Experimentally, a valuable technique for identifying hot spots is through site-directed mutagenesis like alanine scanning, where interface residues are systematically replaced with alanine. The change in binding free energy (ΔΔG) is calculated. Normally, if the ΔΔG >= 2.0 kcal/mol, the residues are defined as hot spots and others are non-hot spots. Two widely used databases are Alanine Scanning Energetics Database (ASEdb)^4^ and Binding Interface Database (BID)^5^, which collected experimental hot spots from Alanine scanning mutagenesis experiments. Recently, there are several new integrated datasets, such as Assi *et al*.’s Ab+ data^6^, SKEMPI database^7^ and Petukh *et al*.’s Alexov_sDB^8^.

Discriminative features for identifying hot spots have been extensively investigated. Analysis of hot spots has discovered that some residues are more favorable in amino acid composition. The most frequent ones, tryptophan (21%), arginine (13.1%) and tyrosine (12.3%), are vital due to their size and conformation in hot spots^9^. Bogan and Thorn find that hot spots are surrounded by energetically less important residues that shape like an O-ring to occlude bulk solvent from the hot spots. A “double water exclusion” hypothesis was proposed to refine the O-ring theory and provide a roadmap for understanding the binding affinity of protein interactions^10^. Besides, some studies show that the hot spots are more conserved than non-hot spots by using sequential and structural analysis^11,12^. Other features are also found that can be used for identifying hot spots, such as pairing potential^13^ or Side chain energy score^14,15^.

Hot spot information from wet-experiments studies is limited because the methods like alanine scanning mutagenesis are costly and time-consuming. Therefore, there is a need for computational approaches to identify hot spots^16^. In general, these methods can be groupded into three main types: molecular dynamics simulations, knowledge-based approaches and machine-learning approaches. Molecular dynamics simulations can offer a detailed analysis of protein interfaces at the atomic level and estimate the changes in binding free energy (ΔΔG). Although some molecular simulation methods provide good predictive results^17–19^, they are not applicable, in practice, for large-scale hot spot predictions due to their huge computational cost. Knowledge-based approaches, such as Robetta^20^ and FOLDEF^21^, which make predictions based on an estimate of the energetic contribution to binding for every interface residue, provide an alternative approach to predict hot spots with much less computational cost.

On the other hand, the machine-learning approaches try to learn the complicated relationship between hot spots and various of residue features and then distinguish hot spots from the interface residues. Ofran and Rost^22^ used neural networks to identify hot spots with features extracted from sequence environment and evolutionary profile of interface residues. Darnell *et al*.^23,24^ introduced two hot spot models by using decision trees to identify hot spots with features such as specificity, FADE points, generic atomic contacts and hydrogen bonds. When the two models were combined, the combined model achieved better predictive accuracy than alanine scanning. Tuncbag *et al*.^13,25^ introduced an effective empirical method by combining solvent accessible surface areas and pair potentials. Cho *et al*.^26^ used a support vector machines (SVM) to identify hot spots with several new features such as the weighted atom packing density, relative accessible surface area and weighted hydrophobicity. Assi *et al*.^6^ presented a probabilistic method that combines features extracted from three main information sources, namely energetic, structural and evolutionary information by using Bayesian Networks (BNs). Lise *et al*.^27^ applied SVMs to predict hot spot residues with features extracted from the basic energetic terms that contribute to hot spot interactions. Xia *et al*.^28^ used SVM classifiers with features such as protrusion index, solvent accessibility. Zhu and Mitchell^29^ proposed two hot spot prediction methods by using SVMs with features like interface solvation, atomic density and plasticity. Wang *et al*.^30^ employed a random forest (RF) to predict hot spots with features from target residues, intra-contact residues and mirror-contact residues. Xia *et al*.^31^ used SVMs to predict hot spots in protein interfaces with features extracted from the sequence, structural and neighborhood features. Moreira *et al*.^32^ presented a web server (SpotOn) to accurately identify hot spots using an ensemble machine learning approach with up-sampling of the minor class. Recently, Qiao *et al*.^33^ proposed a hot spot prediction model by using a hybrid feature selection strategy and SVM classifiers. Our previous method PredHS^15,34^ used SVMs and combined three main information sources, namely site, Euclidean neighborhood and Voronoi neighborhood features, to boost the hot spot prediction performance.

**Figure 1:**
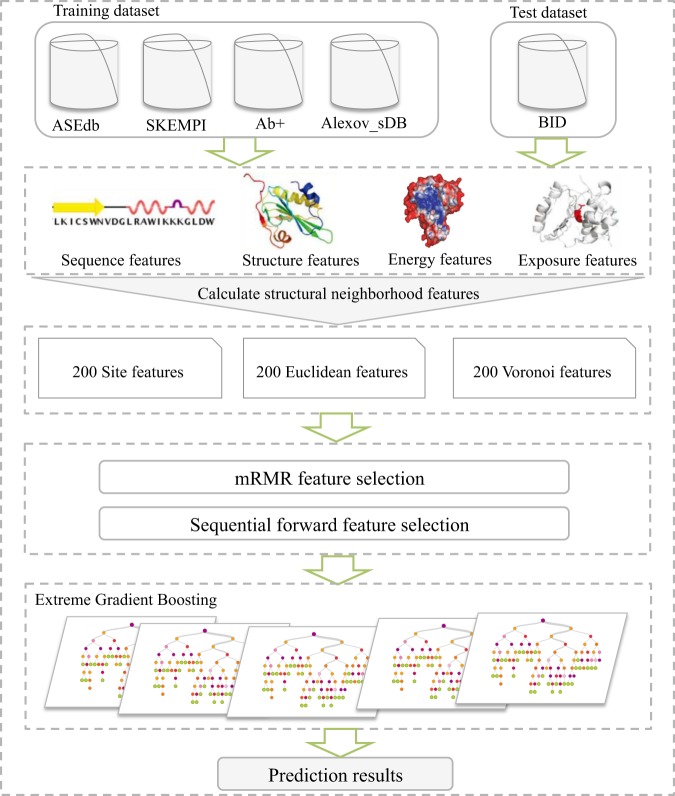
Flowchart of PredHS2. Firstly, the training dataset is generated by integrating four datasets including ASEdb, SKEMPI, Ab+ and Alexov_sDB. And the independent dataset is extracted from the BID database. The residues in the datasets are encoded using a large number of sequence, structure, energy and exposure features and two categories of structural neighborhood properties (Euclidean and Voronoi). As a result, a total of 200 site features, 200 Euclidean features and 200 Voronoi features are obtained. Then a two-step feature selection approach is applied to select the optimal feature set. Finally, the prediction classifier is built using Extreme Gradient Boosting based on the optimal feature set.

In this article, we describe an efficient approach for identifying hot spots at protein-protein interfaces, PredHS2, which is based on our previous PredHS method. First, we generate a new training dataset by integrating several new mutagenesis datasets. Then, we extract a large number of features, especially some novel features, such as solvent exposure features, second structure features and disorder scores. Similar to PredHS’s work, we also use two categories of structural neighborhood properties to better describe the environment around the target site. In all, a wide variety of 600 features are extracted. Next, we apply a new two-step feature selection method to remove redundancy and irrelevant features and then we select a set of 26 optimal features. Finally, we build the PredHS2 model using Extreme Gradient Boosting (XGBoost) and the selected 26 features. We evaluate the performance of our model both on the training dataset and independent test set (BID) and find that PredHS2 significantly outperforms other machine learning algorithms and the existing hot spot prediction methods. The flowchart of PredHS2 is shown in Fig. 1.

## Results

### Performance evaluation

To assess the performance of our prediction model, we adopt 10-fold cross-validation as well as some commonly used measures, such as specificity (SPE), precision (PRE), sensitivity (SEN/Recall), accuracy (ACC), F1-score (F1) and Matthews correlation coefficient(MCC). These measures are calculated as,

1 $$SPE=\frac{TN}{TN+FP}$$

2 $$PRE=\frac{TP}{TP+FP}$$

3 $$SEN=\frac{TP}{TP+FN}$$

4 $$ACC=\frac{TP+TN}{TP+TN+FP+FN}$$

5 $$F1=\frac{2\times Recall\times Precision}{Recall+Precision}$$

6 $$MCC=\frac{TP\times TN-FP\times FN}{\sqrt{(TP+FP)(TP+FN)(TN+FP)(TN+FN)}}$$

where TP, TN, FP and FN represent the numbers of true positive, true negative, false positive and false negative residues in the prediction, respectively. Moreover, Receiver Operating Characteristic (ROC) curve is applied to evaluate the prediction performance, which plots true-positive rate (TPR, sensitivity) versus false-positive rate (FPR, 1-specificity). We also calculate the area under the ROC curve (AUC).

### Feature selection

Features are critical in constructing a classifier using machine learning approaches. In our study, we extract sequence features, structure features, energy features and exposure features, together with Euclidean neighborhood and Voronoi neighborhood properties, for hot spots identification. In total, we generate 600 features, including 200 site properties, 200 Euclidian neighborhood properties and 200 Voronoi neighborhood properties.

**Figure 2:**
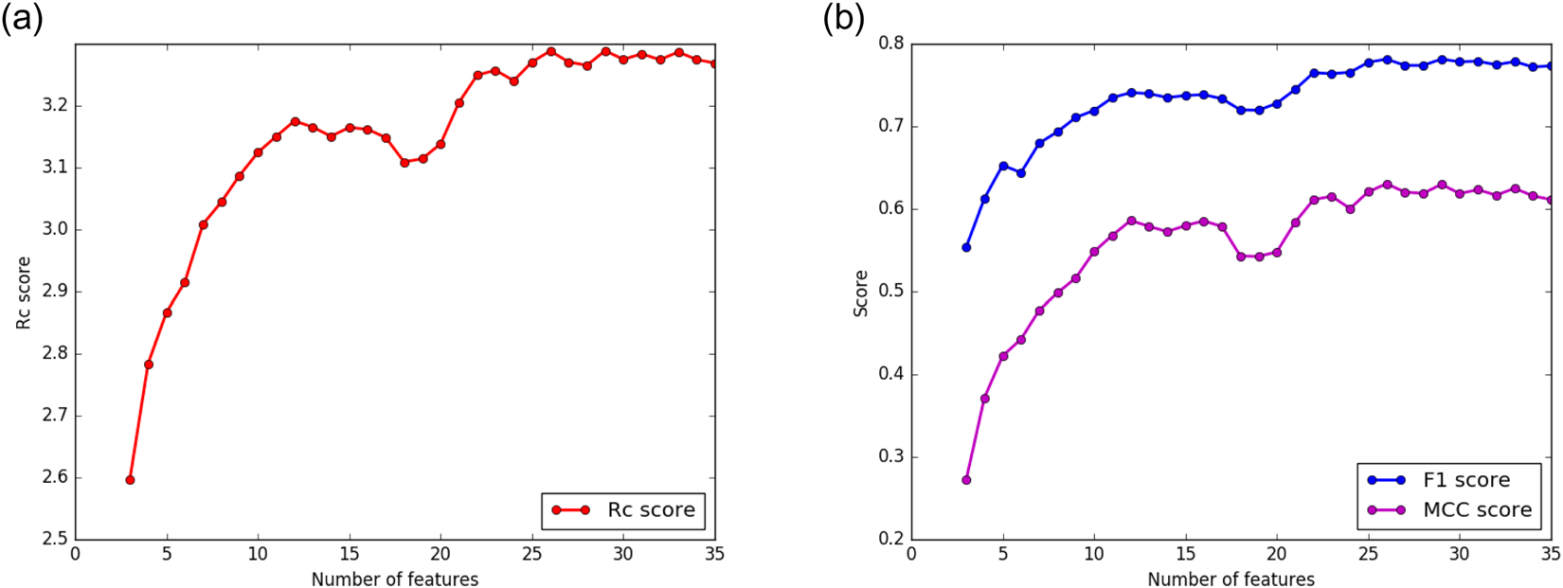
Performance of the two-step feature selection. (**a**) Shows the *R*_*c*_ scores of the top-K features and (**b**) shows the F1 and MCC scores of the top-K features.

To evaluate the feature importance of the 600 candidate properties, we apply a new two-step feature selection method on the training dataset. In the first step, we use minimum Redundancy Maximum Relevance (mRMR)^35,36^ to sort the features. Then we use a wrapper method, where the features are evaluated by 10-fold cross-validation with the XGBoost^37^ algorithm. We select three features from the top-50 features as the initial feature combination, which is similar to the process in HEP^31^. Then we add correlation features by using sequential forward selection (SFS)^38^ method. In the SFS method, features are sequentially added to the initial feature combinations till an optimal feature subset is acquired. Each added feature is the one whose add maximizes the performance of the classifier. The ranking criterion *R*_*c*_ indicates the prediction performance of the classifier, which is used in our previous PredHS^15^ and defined in the Methods section. This step-by-step feature selection method continues until the *R*_*c*_ score no longer increased. Figure 2 shows the *R*_*c*_, F1 and MCC scores of the top-K features. Consequently, we select a set of 26 optimal features.

To illustrate the necessity for feature selection, Firstly, we get the predictive performance (F1 = 0.689) when we use all the features. Then, we compare the two-step feature selection method with three extensively used feature selection methods, including random forest (RF)^39^, recursive feature elimination (RFE)^40^ and maximum relevance minimum redundancy (mRMR)^35^. Table 1 displays the prediction performance of the four feature selection methods based on the training dataset with 10-fold cross-validation. Table 1 shows that feature selection can improve the performance of a classifier in our study. After feature selection, there is at least 6% increase in F1-score. Table 1 also shows that the two-step feature selection method gets the highest F1 score. The result illustrates that our two-step feature selection algorithm can efficiently boost the prediction performance with lower computational cost and less risk of overfitting.

**Table 1:** The performance of the two-step feature selection method in comparison with other feature selection methods.

Method	ACC	SPE	PRE	SEN	F1	MCC
All features	0.753	0.806	0.721	0.677	0.689	0.487
RF	0.808	0.862	0.799	0.722	0.756	0.598
RFE	0.811	0.846	0.809	0.769	0.774	0.626
mRMR	0.794	0.826	0.769	0.763	0.757	0.588
Two-step	0.818	0.844	0.786	0.783	0.782	0.63

### Assessment of feature importance

**Figure 3:**
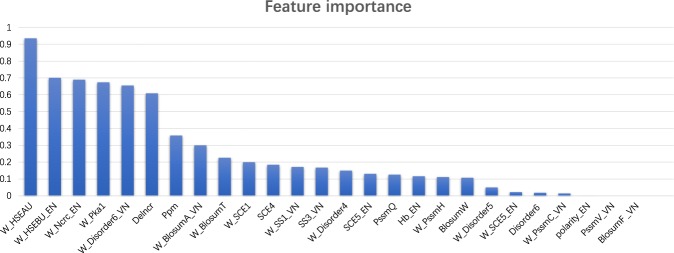
The feature importance of the selected 26 features.

To better access the importance of the selected 26 features, we calculate the F-scores based on the training dataset. F-score can measure the discriminative power of individual features between hot spots and non-hot spots^28^. Figure 3 displays the feature importance of the selected 26 features and their contribution to the identification ability (in descending order). Table 2 lists the detailed information about the optimal 26 features, which are ranked by their F-scores.

**Table 2:** The optimal 26 features for identifying hot spots based on the two-step feature selection method.

Rank	Feature name	Symbol	F-score	Feature type
1	Weighted Solvent exposure features (HSEAU)	W_HSEAU	0.9346	Site
2	Weighted Solvent exposure features (HSEBU) in Euclidean neighborhood	W_HSEBU_EN	0.7007	Euclidian
3	Weighted normalized residue contacts in complex in Euclidean neighborhood	W_Ncrc_EN	0.6894	Euclidian
4	Weighted Side-chain environment (pKa_1)	W_Pka1	0.6737	Site
5	Weighted Disorder_6 score in Voronoi neighborhood	W_Disorder6_VN	0.6546	Voronoi
6	Δ(delta) normalized residue contacts	Delncr	0.6086	Site
7	Pair potentials in monomer	Ppm	0.3576	Site
8	Weighted Blosum (A) in Voronoi neighborhood	W_BlosumA_VN	0.2991	Voronoi
9	Weighted Blosum (T)	W_BlosumT	0.2258	Site
10	Weighted Sidechain energy score	W_SCE1	0.1991	Voronoi
11	Side chain energy score (SCE-score (conserv))	SCE4	0.1842	Site
12	Weighted Second Structure (SS) helix in Voronoi neighborhood	W_SS1_VN	0.1716	Voronoi
13	Second Structure (SS) coil in Voronoi neighborhood	SS3_VN	0.1675	Voronoi
14	Weighted Disorder_4 score	W_Disorder4	0.1502	Site
15	SCE-score (conbine_1) in Euclidean neighborhood	SCE5_EN	0.13108	Euclidian
16	PSSM (Q)	PssmQ	0.1261	Site
17	Hydrogen bonds in Euclidean neighborhood	Hb_EN	0.1163	Euclidian
18	Weighted PSSM (H)	W_PssmH	0.1117	Site
19	Blosum (W)	BlosumW	0.1078	Site
20	Weighted Disorder_5 score	W_Disorder5	0.0502	Site
21	Weighted SCE-score (conbine_1) in Euclidean neighborhood	W_SCE5_EN	0.02217	Euclidian
22	Disorder_6 score	Disorder6	0.01865	Site
23	Weighted PSSM (C) in Voronoi neighborhood	W_PssmC_VN	0.01427	Voronoi
24	Physicochemical properties (polarity) in Euclidean neighborhood	polarity_EN	0.001	Euclidian
25	PSSM (V) in Voronoi neighborhood	PssmV_VN	0.00069	Voronoi
26	Blosum (F) in Voronoi neighborhood	BlosumF_VN	0.00018	Voronoi

As shown in Fig. 3 and Table 2, the weighted solvent exposure features (HSEAU) and weighted solvent exposure features(HSEBU) in Euclidean neighborhood achieve the highest scores, which means that solvent exposure features have better discriminative power than traditional sequence and structural features in identifying hot spots. The weighted normalized residue contacts in the complex in Euclidean neighborhood shows good discriminative power with the F-score of 0.689. The weighted Side-chain environment (pKa_1) and weighted Disorder_6 score in Voronoi neighborhood are newly added features and they also achieve high scores. Through the data statistics of the 26 optimal features in Table 2, the newly added features account for 13 out of the total 26 optimal features, such as solvent exposure features, disorder score, blocks substitution matrix and hydrogen bonds. It means that the newly added features in PredHS2 compared with the original PredHS are highly effective. There are 12 site properties and 6 Euclidian neighborhood properties and 8 Voronoi neighborhood properties in the total 26 optimal features, which means that the structural neighborhood properties contribute to identifying hot spots, which is consistent with the findings in PredHS. As reported in the previous method, the ASA-based features have good discriminative power. Although there are no ASA-based features in the selected 26 features, there are 14 features with weighted which are related to the Weighted fraction buried, this means that the Weighted fraction buried and the features related to ASA are also important.

To further state how features are shown to be more or less important, we use a heuristic for correcting biased measures of feature importance, called permutation importance (PIMP)^41^. The method normalizes the biased measure based on a permutation test and returns significance P-values for each feature. The PIMP P-values are easier to interpret and provide a common measure that can be used to compare feature relevance among different models. As shown in the supplementary material (Table S1), we can find that the PIMP P-value of the majority features are less than 0.05, which means that the majority of 26 optimal features are significant.

**Figure 4:**
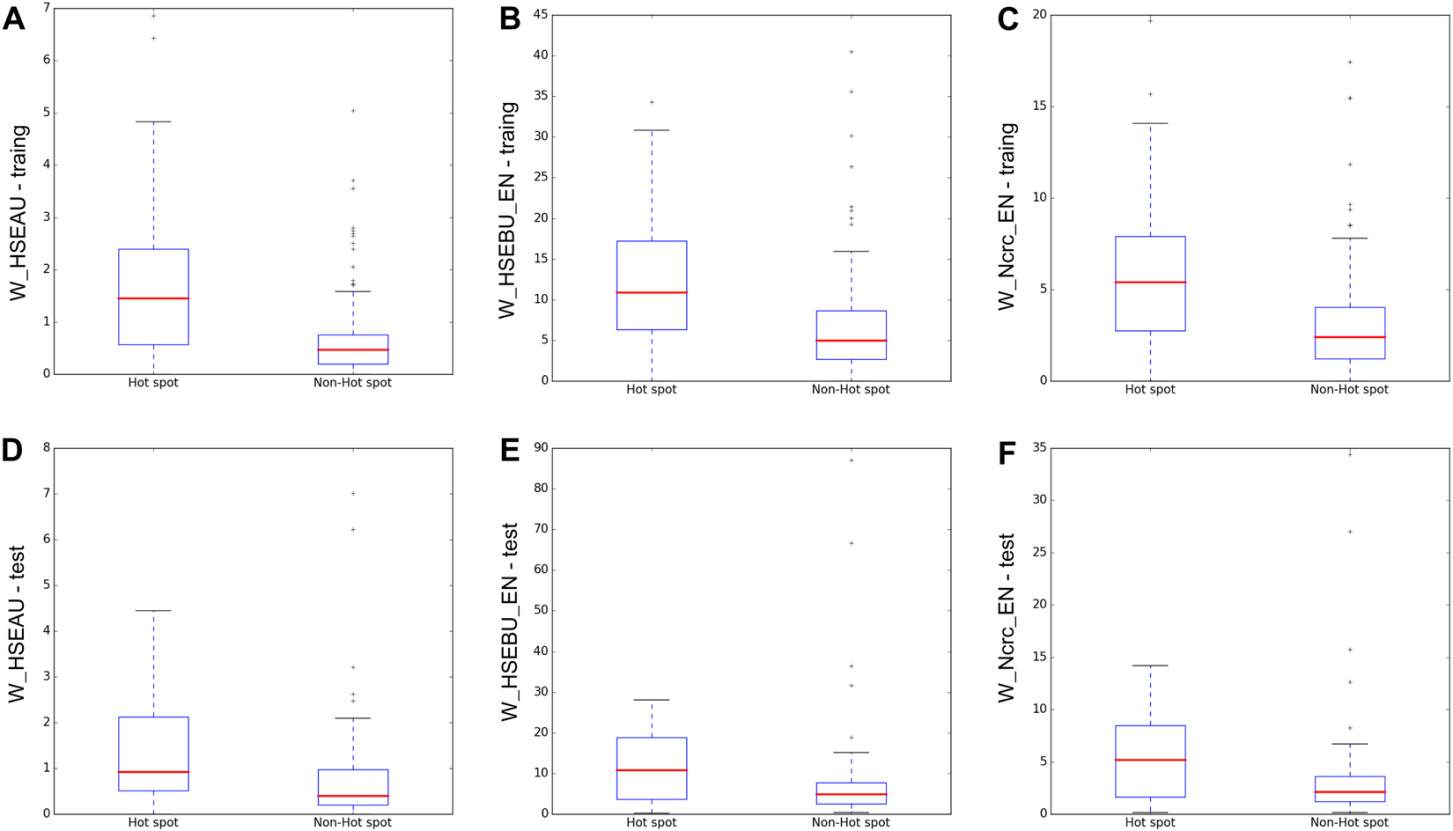
Box plot of hot spots and non-hot spots concerning their W_HSEAU (**A**), W_HSEBU_EN (**B**) and W_Ncrc_EN (**C**) in training dataset and W_HSEAU (**D**), W_HSEBU_EN (**E**) and W_Ncrc_EN (**F**) in test dataset, respectively. In each box plot, the bottom and top are severally the lower and upper quartiles and the middle line of the box is the median.

Here, we choose the top-3 features of the optimal 26 features for detail analysis. To display the discriminative power of the top-3 features for distinguishing hot spots from non-hot spots, we employ the box plot and F-test which is available in scikit-learn^42^. As shown in Fig. 4, the discriminative power of the top-3 features between hot spots and non-hot spots are prominent. Figure 4A shows the box plot of W_HSEAU in the training dataset. The median value of W_HSEAU of hot spots is 1.44, while the median value of non-hot spots is 0.47, with P-value = 4.91 × 10^−15^. Figure 4B is the box plot of W_HSEBU_EN, in which the median value of W_HSEBU_EN of hot spots (10.9) is higher than that of non-hot spots (4.98), with P-value = 6.91 × 10^−12^. These results suggest the hot spots have a higher solvent exposure values^43^ than non-hot spots. Figure 4C represents the box plot of weighted normalized residue contacts in the complex in Euclidean neighborhood (W_Ncrc_EN). The median W_Ncrc_EN of hot spots is 5.4 and that of non-hot spots is 2.39 (P-value = 9.9 × 10^−12^). Thus, W_Ncrc_EN is a significant feature for distinguishing hot spots from non-hot spots. In our previous work (PredHS), we also found the features related to residue contacts were important. Besides, Fig. 4D–F show the box plots of the three features between hot spots and non-hot spots in the independent test set. We also find that these features have high discriminative power.

### Comparison with other machine learning methods

PredHS2 uses XGBoost^37^ to build the final model with the 26 optimal features. In this section, we compare PredHS2 with Support Vector Machines (SVM)^44,45^, Random Forest (RF)^46^, gradient tree boosting (GTB)^47^ and Multi-layer Perceptron (MLP) classifier^48,49^ which are known to perform relatively well on variety tasks. All these algorithms are implemented using the scikit-learn^42^ python libraries with the default parameter configuration. Table 3 shows the performance comparison of PredHS2 and other machine learning methods on the training dataset with 10-fold cross-validation. It can be seen that PredHS2, RF, SVM, GTB and MLP achieve F1 score of 0.782, 0.597, 0.621, 0.709 and 0.600, respectively. The F1 score is the harmonic mean of the precision and sensitivity, which is extensively used to deal with unbalanced data. PredHS2 also outperforms the other four machine learning methods in other performance metrics. The results indicate that our proposed XGBoost-based PredHS2 model can boost the prediction performance.

**Table 3:** Comparison with other machine learning methods on the training dataset with 10-fold cross-validation.

Method	ACC	SPE	PRE	SEN	F1	MCC
RF	0.700	0.827	0.695	0.528	0.597	0.377
SVM	0.702	0.789	0.674	0.587	0.621	0.388
GTB	0.761	0.800	0.717	0.709	0.709	0.510
MLP	0.648	0.655	0.603	0.640	0.600	0.306
PredHS2	0.818	0.844	0.786	0.783	0.782	0.630

### Comparison with existing state-of-the-art methods

To further evaluate the performance of the proposed PredHS2, ten existing state-of-the-art protein-protein hot spots prediction methods, including iPPHOT^33^, HEP^31^, PredHS^15^, APIS^28^, Robetta^20^, FOLDEF^21^, KFC^23^, MINERVA^26^, KFC2a and KFC2b^29^, are compared on the independent test dataset.

Table 4 describes the detailed results. The prediction results of iPPHOT are obtained from the iPPHOT web server^33^. The results of PredHS are obtained from the PredHS web server^34^. The results of other methods are extracted from the summarized data in HEP^31^. Our PredHS2 method shows the best predictive performance (accuracy = 0.87, sensitivity = 0.77. specificity = 0.92, precision = 0.81, F1 = 0.79 and MCC = 0.70). This indicate that 77% of the true hot spots are rightly predicted (sensitivity) and 92% of the non-hot spots are rightly predicted (specificity). iPPHOT and HEP have a better sensitivity of 0.79 and 0.84, respectively. PredHS have a better specificity of 0.93. We can see that our PredHS2 method substantially outperforms the existing methods in four performance metrics (accuracy, precision, F1-score and MCC). PredHS2 achieves the highest F1-score of 0.79, which means PredHS2 has a better balance between sensitivity and specificity. PredHS2 obtains at least 9% increase in F1-score and 13% increase in MCC value.

**Table 4:** Performance comparison of PredHS2 and other existing methods on the independent test dataset.

Method	TP	TN	FP	FN	ACC	SPE	PRE	SEN	F1	MCC
PredHS2	30	80	7	9	0.87	0.92	0.81	0.77	0.79	0.70
iPPHOT	31	51	36	8	0.65	0.59	0.46	0.79	0.58	0.35
HEP	32	68	21	6	0.79	0.76	0.60	0.84	0.70	0.56
PredHS-SVM	23	81	6	16	0.83	0.93	0.79	0.59	0.68	0.57
APIS	28	67	21	11	0.75	0.76	0.57	0.72	0.64	0.45
Robetta	12	80	11	24	0.72	0.88	0.52	0.33	0.41	0.25
FOLDEF	10	78	11	28	0.69	0.88	0.48	0.26	0.34	0.17
KFC	12	75	12	27	0.69	0.85	0.48	0.31	0.38	0.19
MINERVA	17	79	9	22	0.76	0.9	0.65	0.44	0.52	0.38
KFC2a	29	64	24	10	0.73	0.73	0.55	0.74	0.63	0.44
KFC2b	21	77	12	17	0.77	0.87	0.65	0.55	0.60	0.44

**Figure 5:**
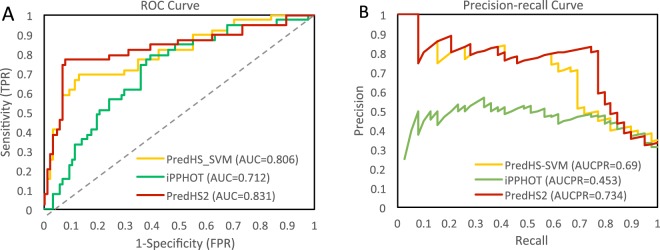
Comparison of PredHS2, iPPHOT and PredHS-SVM methods on the independent test dataset. (**A**) is the ROC curves; (**B**) is the Precision-Recall curves.

Figure 5 shows the comparison of PredHS2, iPPHOT and PredHS-SVM methods on the independent test dataset. Figure 5A shows the ROC curves and AUC (ROC) scores, PredHS2, iPPHOT and PredHS-SVM achieve AUC (ROC) scores of 0.831, 0.712 and 0.806, respectively. Figure 5B shows the Precision-Recall curves. It can be seen that PredHS2, iPPHOT and PredHS-SVM achieve AUC (Precision-Recall curve) of 0.734, 0.453 and 0.69, respectively. According to these results, our PredHS2 achieves the best predictive performance.

### Case study

**Figure 6:**
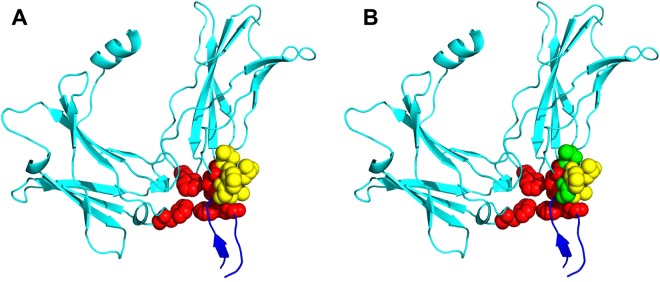
Hot spot prediction results by using PredHS2 (**A**) and iPPHOT (**B**) for the EPO receptor complex. True positives (red), true negatives (yellow), false positives (green) and false negatives (purple) are colored. Chain A is colored in cyan and chain C is colored in blue.

We describe a case study of applying PredHS2 to predict hot spots from the complex of erythropoietin (EPO) receptor (PDB ID:1EBP, chain A) and erythropoietin mimetic peptide (PDB ID: 1EBP, chain C). As shown in Fig. 6, four hot spots (PHE93:A, PHE205:A, MET150:A and TRP13:C) and five non-hot spots have been experimentally determined at the binding interface. We use the following color scheme to display the results: true positives are colored in red; true negatives are colored in yellow; false positives are colored in green; false negatives are colored in purple. For the nine alanine-mutated residues, iPPHOT correctly predicted the four hot spots but incorrectly predicted two non-hot spots (THR151:A, GLY9:C) as hot spots. In contrast, our PredHS2 approach correctly predicted all the nine residues: four residues (PHE93:A, PHE205:A, MET150:A and TRP13:C) are identified as hot spots and the rest as non-hot spots.

## Conclusion

We have shown that PredHS2, a powerful computational framework, can reliably predict hot spots at the protein-protein binding interface. PredHS2 combines a variety of sequence, structure, energy, exposure and other features and together with Euclidean and Voronoi neighborhood properties, to improve prediction of hot spots, which relies on a two-step feature selection algorithm to select the most useful and contributive features to build the prediction classifiers. We also investigated what information of residue micro-environments is relevant and essential to the prediction of hot spots. Benchmarking experiments showed that our PredHS2 approach has significantly outperformed the other existing state-of-the-art methods on both benchmark and independent test datasets. In summary, the performance improvement benefits from the following aspects: (1) construction of a high-quality non-redundant training dataset; (2) integration of a variety of features especially two categories of structural neighborhood properties that collectively make a useful contribution to the performance; (3) a two-step feature selection approach to retrieve the useful features; (4) the XGBoost algorithm to effectively build the prediction model.

We believe that PredHS2 can be an effective tool for accurately predicting protein-protein biding hot spots with the increasing availability of high-quality structure data. A web server implementation is freely available at http://predhs2.denglab.org.

## Methods

### Datasets

In the previous study, a widely used training dataset is the work of Cho *et al*.^26^, which was obtained from ASEdb^4^ and the published data of Kortemme and Baker^20^. It consists of 265 experimentally mutated interface residues extracted from 17 protein-protein complexes. Recently, there are several new integrated databases in the published literatures, such as Assi *et al*.‘s Ab+ data^6^, SKEMPI database^7^ and Petukh *et al*.‘s Alexov_sDB^8^.

In this work, we construct a new training dataset of 313 alanine-mutated interface residues extracted from 34 protein complexes after redundancy removal. The dataset is extracted from four datasets including Alanine Scanning Energetics (ASEdb)^4^, SKEMPI database^7^, Assi *et al*.‘s Ab+ data^6^ and Petukh *et al*.‘s Alexov_sDB^8^. We merge the above datasets and exclude the protein complexes in the BID dataset^5^. A total of 71 unique protein-protein complexes are obtained. Then we use CD-HIT^50^ to remove the redundancy and obtain a benchmark of 34 protein complexes. The interface residues are defined as hot spots with the ΔΔG >= 2.0 kcal/mol and the others are defined as non-hot spots. As a result, the benchmark has 313 interface residues of which contains 133 hot spots residues and 180 non-hot spots residues. The benchmark can be found in Supplemental File 1.

Similar to our previous PredHS, we use the BID database^5^ as the independent test set to further assess the performance of our model. In the BID database, the alanine mutation data were labeled as “strong”, “intermediate”, “weak”, or “insignificant”. In this study, only “strong” mutations are considered as hot spots and others are non-hot spots. Furthermore, the proteins in this independent test set are non-homologous to those proteins in the above training dataset. The test dataset is a collection of 18 complexes contained 127 alanine-mutated residues, where 39 interface residues are hot spots. The data are listed in Supplemental File 2.

### Features representation

Features for machine learning methods is an important factor in building a model. Based on previous studies, we investigate a large number of features for identifying hot spots. We first extract 100D site features including exposure, energy, sequences and structure features. And then we calculate Euclidean neighborhood and Voronoi neighborhood features for each amino acid, which is similar to our previous PredHS^15^. For site features, a wide variety of exposure, energy, sequence and structure properties are selected for predicting hot spots in protein-protein iteraction, including physicochemical properties (12 features)^51^, Side-chain environment (pKa) (2 features)^52^, Position specific score matrix (PSSM) (20 features)^53^, Evolutionary conservation score (C-score) (1 feature)^54^, Solvent accessible area (ASA) (6 features)^55,56^, Normalized atom contacts and normalized residue contacts (6 features)^15^, Pair potentials (3 features)^13,57^, Topographical score (TOP) (1 features)^6^, Four-body pseudo-potential (1 features)^14^, Side chain energy score (6 features)^14^, Local structural entropy(LSE)(3 features)^58^, Nearby interface score (1 features), Voronoi contacts (2 features)^59^, Second Structure (SS) (3 features), Disorder score (6 features)^60^, Blocks substitution matrix(Blosum62)(20 features)^61^, Solvent exposure features (7 features), Conservation score (1 feature), Hydrogen bonds (Hbplus) (1 feature).

In total, a large number of 100 × 3 × 2 = 600 features are selected for identifying hot spots residues. Among these features, 324 features are used in our previous PredHS^15^ and the rest are newly added to PredHS2. The details about these novel features are described below.

#### Physicochemical properties

The eleven physicochemical properties of an amino acid are hydrophobicity, hydrophilicity, polarity, polarizability, propensities, average accessible surface area, Number of atoms, number of electrostatic charges (NEC), number of potential hydrogen bonds (NPHB), molecular mass, electron-ion interaction pseudopotential (EIIP). The original values of the eleven physicochemical attributes for each residue are obtained from the AAindex database^51^. Besides, we also used pseudo hydrophobicity (PSHP) defined in HEP^31^ method.

#### Side-chain environment (pKa)

The Side-chain environment (pKa) is an effective metric in determining environmental characteristics of a protein. The value of pKa is obtained from Nelson and Cox^52^ representing protein side-chain environmental factor and is extensively used by previous studies^62^.

#### Second Structure (SS)

The secondary structure is a significant structure-based attribute for prediction of hot spots in protein interface, which is computed by DSSP^55^. It is divided into three different categories namely helix, sheet and coil. In our study, types G, H and I in DSSP secondary structure are regarded as the helix; types B and E are considered as the sheet; and types T, S and blank are recognized as the coil. Therefore, secondary structure of each residue is encoded as a three-dimensional vector: helix (1, 0, 0), sheet (0, 1, 0) or coil (0, 0, 1).

#### Disorder score

We used DISOPRED^63^ and DisEMBL^64^ to predict dynamically disordered regions of amino acid in the protein sequence. Disorder score is proved to be an is effective feature by previous studies^62,65^.

#### Blocks substitution matrix

Blosum62^61^ is a substitution matrix which can be used for proteins sequence alignment. We use Blosum62 to count the relative frequencies of amino acid and their substitution probabilities.

#### Solvent exposure features

Half-sphere exposure (HSE) is an excellent measure of solvent exposure, HSE has a superior performance concerning protein stability, conservation among fold homologs, computational speed and accuracy^43^. HSE conceptually separates an amino acid’ sphere into two half-spheres: HSE-up corresponds to the upper sphere in the direction of the chain side of the residue, while HSE-down points to the lower sphere in the direction of the opposite side^66^. In other words, a residue’s HSE-up measure is defined as the number of *C*_*α*_ atoms in its upper half-sphere, which contains the *C*_*α*_ − *C*_*β*_ vector. Similarly, HSE-down is defined as the number of *C*_*α*_ atoms in the other lower half-sphere^66^. HSEpred^66^ is used to facilitate the HSE and CN (coordination number) prediction. Based on protein structure, We employ hsexpo^43^ to compute the exposure features, such as HSEAU (number of *C*_*α*_ atoms in the upper sphere), HEAD(number of *C*_*α*_ atoms in the lower sphere), HSEBU (the number of *C*_*β*_ atoms in the upper sphere), HSEBD(the number of *C*_*β*_ atoms in the lower half sphere), CN (coordination number), RD (residue depth) and RDa (*C*_*α*_ atom depth).

#### Conservation score

The Conservation score is a sequence-based feature, it expresses the variability of residues at each position in the protein sequence. it is calculated based on PSSM^53^ and is defined as follows:

7 $$Scor{e}_{i}=-\,\sum _{j=1}^{20}\,{p}_{i,j}lo{g}_{2}{p}_{i,j}$$

where *p*_*i*, *j*_ represents the frequency of residue j at position i. If a residue has a lower conservation score, this means the residue has a lower entropy (more conserved).

#### Hydrogen bonds

We calculate the number of Hydrogen bonds by using HBPLUS^67^.

#### Weighted fraction buried

As same as the procedure in PredHS, conventional structure-related features such as solvent accessible area and surface area burial (ΔASA) are highly effective to predict hot spots^26^. To improve discrimination performance, the Weighted fraction buried (*W*_*FB*_) for residue i is calculated by weighting the ratio of surface area burial (ΔASA) to the solvent accessibility in the monomer as below:

8 $${W}_{FB}(i)=W(i)\ast \frac{{\rm{\Delta }}AS{A}_{i}}{ASA\,of\,the\,i-th\,residue\,in\,the\,monomer}$$

The *W*(*i*) weights the contribution of each residue according to its relative contribution to the total interface area, it is defined as follows:

9 $$W(i)=\frac{{\rm{\Delta }}AS{A}_{i}}{\sum _{j=1}\,({\rm{\Delta }}AS{A}_{j})}\,,\,j\,stands\,for\,an\,interface\,residue$$

#### Structural Neighborhood properties

Similar to our previous work in PredHS, we use Euclidean distance and Voronoi diagram to calculate two types of structural neighborhood properties. The Euclidean neighborhood is a set of residues which located within a sphere of 5 Å defined by the minimum Euclidean distances between any heavy atoms of the surrounding residues and any heavy atoms from the central residue. Besides, We use Voronoi diagram/Delaunay triangulation to define neighbor residues in 3D protein structures. Voronoi tessellation partitions the 3D space of protein structures into Voronoi polyhedra around individual atoms. In the circumstances of Voronoi diagram/Delaunay triangulation, a pair of residues is considered to be neighbors when at least one pair of heavy atoms of each residue has a Voronoi facet in common (in the same Delaunay tetrahedra). We used the Qhull package^68^ to calculate Voronoi/Delaunay polyhedra.

### Two-step feature selection

Feature selection is performed to remove redundancy and irrelevant features, which contribute to further improving the performance of a classifier. Based on the 600 candidate properties, we apply a new two-step feature selection approach to select the most important features for identifying hot spots.

In the first step, we evaluate the feature elements using minimum Redundancy Maximum Relevance (mRMR)^35^. Max-Relevance means that selecting the features with the highest relevance to the target variable, while Min-Redundancy means that selecting the candidate features with minimal redundancy to the features already selected. The relevance and redundancy in mRMR are measured by the mutual information(MI), which is defined as:

10 $$I(x,y)=\iint p(x,y)\mathrm{log}\,\frac{p(x,y)}{p(x)p(y)}dxdy$$

where *x* and *y* are two random variables, *p*(*x*), *p*(*y*) and *p*(*x*, *y*) are their probabilistic density functions. By using the mRMR method, we get the Top-50 features and Top-500 features.

In the second step, we use a wrapper-based feature selection. The features are evaluated by 10-fold cross-validation with the XGBoost^37^ algorithm. We first select three features from the Top-50 features as the initial feature combinations, which is similar to the process in HEP^31^. Then we add correlation features by using sequential forward selection (SFS) method^38^. In the SFS method, features from the Top-500 features are sequentially added to the initial feature combinations until the ranking criterion *R*_*c*_ no longer increased. The ranking criterion *R*_*c*_ is used in PredHS^15^ and represent the prediction preformance of the predictor. In each step, we choose the new feature with the highest *R*_*c*_ score. The *R*_*c*_ is defined as follows:

11 $${R}_{c}=\frac{1}{n}\sum _{i=1}^{n}\,\{AC{C}_{i}+SE{N}_{i}+SP{E}_{i}+AU{C}_{i}\}$$

where n is the repeat times of 10-fold cross-validation: *ACC*_*i*_, *SEN*_*i*_, *SPE*_*i*_ and *AUC*_*i*_ represent the values of the accuracy, sensitivity, specificity and AUC score of the i-th 10-fold cross-validation, respectively.

### Extreme Gradient Boosting algorithm

Gradient Boosting algorithm^69^ is a meta-algorithm to construct an ensemble strong learner from weak learners, typically decision trees. The Extreme Gradient Boosting (XGBoost) proposed by Chen and Guestrin^37^ is an efficient and scalable variant of the Gradient Boosting algorithm. In recent years, XGBoost^37^ is used extensively by data scientists and achieves satisfactory results on many machine learning competitions. XGBoost have advantages for its features such as ease of use, ease of parallelization and high predictive accuracy.

In this study, the prediction of hot spots in protein interfaces can be considered as a binary classification problem. For the given input feature vectors *χ*_*i*_ (*χ*_*i*_ = {*x*_1_, *x*_2_, …, *x*_*n*_}, *i* = 1, 2, …, *N*), we use XGBoost to predict the class label *y*_*i*_ (*y*_*i*_ = {−1, +1}, *i* = 1, 2, …, *N*), where ‘−1’ represents non-hot spots residue and ‘+1’ indicate hot spots. And XGBoost is implemented using the scikit-learn^42^ python libraries. In the algorithm, XGBoost is an ensemble of *K* Classification and Regression Trees (CART)^37,70^. Basically, the training procedure is done by using an “additive strategy”: Given a residue i with a vector of descriptors *χ*_*i*_, a tree ensemble model uses *K* additive functions to predict the output.

12 $${\hat{y}}_{i}=\sum _{k=1}^{K}\,{f}_{k}\,({\chi }_{i}),\,{f}_{k}\in F$$

Here *f*_*k*_ represents an independent tree structure with leaf scores and *F* is the space of functions containing all Regression trees. To learn the space of functions used in the model, XGBoost tries to minimize the following regularized objective.

13 $$Obj=\sum \,l({\hat{y}}_{i},{y}_{i})+\sum \,{\rm{\Omega }}(\,{f}_{k})\,,\,where\,{\rm{\Omega }}\,(f)=\gamma T+\frac{1}{2}\lambda {\Vert \omega \Vert }^{2}$$

In the equation above, the first term is a differentiable convex loss function, *l*, which measures the difference between the prediction *ŷ*_*i*_ and the target *y*_*i*_. The second term Ω penalizes the complexity of the model where *T* and *ω* are the number of leaves in the Tree and the score on each leaf respectively. *γ* and *λ* are constants to control the degree of regularization. The regularization term Ω helps to smooth the final learned weights to avoid overfitting. More directly, the regularized objective will tend to select a model adopting simple and predictive functions.

In XGBoost, the loss function is expanded into the second order Taylor expansion to quickly optimize the objective in the general setting, while the L1 and L2 regularizations are introduced. Besides the regularized objective, shrinkage and column (feature) subsampling are two additional techniques used to further reduce overfitting^37,71^. After each step of boosting, shrinkage scales newly added weights by a factor *η*. This reduces the influence of each tree and makes the model learn slowly and (hopefully) better. Column subsampling is commonly used in RandomForest^39^. It considers only a random subset of descriptors in building a given tree. The usage of column subsampling also speeds up the training process by reducing the number of descriptors to consider. XGBoost uses the sparsity-aware split finding approach to improve gradient boosting algorithm for handling sparse data, introduces a weighted quantile sketch algorithm for approximate optimization and proposes a column block structure for parallelization.

We use a grid search strategy to select the optimal parameters of XGBoost with 10-fold cross-validation on the benchmark dataset. The optimized number of boosted trees of the XGBoost is 2000 and the maximum tree depth for base learners (max_depth) is 5 and gamma is 0.005. The rest use the default parameters.

### The PredHS2 method

Figure 1 shows the overview of the PredHS2 architecture. Firstly, we construct a new training dataset of 313 alanine-mutated interface residues extracted from 34 protein complexes. The dataset is generated from four datasets, including four datasets including ASEdb, SKEMPI, Ab+ and Alexov_sDB. Then, we extract various features from exposure, energy, sequence and structure features, together with Euclidean neighborhood and Voronoi neighborhood properties. In total, we generate 600 features for hot spots identification. Among these features, there are 324 features which are used in our previous PredHS. Meanwhile, we add some novel effective features to PredHS2, such as solvent exposure features, side-chain environment, the second structure, disorder score and block substitution matrix. Next, we apply a new two-step feature selection method to remove redundancy and irrelevant features. In the first step, we evaluated the feature elements using minimum Redundancy Maximum Relevance (mRMR) and we get the Top-50 features and Top-500 features. In the second step, we use a wrapper-based feature selection, where the features are evaluated by 10-fold cross-validation with the XGBoost algorithm. We first select three features from the Top-50 features as the initial feature combinations. Then we add correlation features by using sequential forward selection (SFS) method. In the SFS method, we choose the new feature from Top-500 features with the highest *R*_*c*_ score in each step. Consequently, we select a set of 26 optimal features. Finally, an Extreme Gradient Boosting (XGBoost) classifier is built to predict hot spots in protein interfaces. We evaluate the performance of our PredHS2 by the 10-fold cross validation on the new training dataset and then we compare our PredHS2 with the previous studies on the independent test set.

The PredHS2 webserver is available at http://predhs2.denglab.org.

## Electronic supplementary material

Supplementary material
