# Supplementary material for: Enhanced Prediction of Hot Spots at Protein-Protein Interfaces Using Extreme Gradient Boosting
Source: Sci Rep. 2018 Sep 24;8:14285. doi: 10.1038/s41598-018-32511-1 (PMC6155324; doi:10.1038/s41598-018-32511-1)
Supplement: Supplementary file 1 — Supplementary material [file 41598_2018_32511_MOESM1_ESM.docx]

Supplementary material

Enhanced Prediction of Hot Spots at Protein-Protein Interfaces Using Extreme Gradient Boosting

Hao Wang, Chuyao Liu and Lei Deng

Table S1 The PIMP P-values of the 26 optimal features.

| **Rank** | **Feature name** | **Symbol** | **PIMP**  **P-value** | **Feature type** |
| --- | --- | --- | --- | --- |
| 1 | Weighted Solvent exposure features (HSEAU) | W_HSEAU | 1.66E-38 | Site |
| 2 | Weighted Solvent exposure features (HSEBU) in Euclidean neighborhood | W_HSEBU_EN | 3.15E-19 | Euclidian |
| 3 | Weighted normalized residue contacts in complex in Euclidean neighborhood | W_Ncrc_EN | 3.81E-31 | Euclidian |
| 4 | Weighted Side-chain environment(pKa_1) | W_Pka1 | 5.13E-07 | Site |
| 5 | Weighted Disorder_6 score in Voronoi neighborhood | W_Disorder6_VN | 1.29E-02 | Voronoi |
| 6 | ∆(delta) normalized residue contacts | Delncr | 9.91E-05 | Site |
| 7 | Pair potentials in monomer | Ppm | 2.89E-02 | Site |
| 8 | Weighted Blosum(A) in Voronoi neighborhood | W_BlosumA_VN | 1.17E-07 | Voronoi |
| 9 | Weighted Blosum(T) | W_BlosumT | 8.23E-10 | Site |
| 10 | Weighted Sidechain energy score | W_SCE1 | 5.93E-02 | Voronoi |
| 11 | Side chain energy score(SCE-score) SCE-score(conserv) | SCE4 | 6.90E-15 | Site |
| 12 | Weighted Second Structure(SS) helix in Voronoi neighborhood | W_SS1_VN | 8.47E-02 | Voronoi |
| 13 | Second Structure(SS) coil in Voronoi neighborhood | SS3_VN | 1.99E-10 | Voronoi |
| 14 | Weighted Disorder 4 score | W_Disorder4 | 3.49E-01 | Site |
| 15 | SCE-score(conbine_1) in Euclidean neighborhood | SCE5_EN | 8.91E-02 | Euclidian |
| 16 | PSSM(Q) | PssmQ | 4.96E-02 | Site |
| 17 | Hydrogen bonds in Euclidean neighborhood | Hb_EN | 2.00E-10 | Euclidian |
| 18 | Weighted PSSM(H) | W_PssmH | 1.01E-13 | Site |
| 19 | Blosum(W) | BlosumW | 1.14E-01 | Site |
| 20 | Weighted Disorder_5 score | W_Disorder5 | 1.09E-01 | Site |
| 21 | Weighted SCE-score(conbine_1) in Euclidean neighborhood | W_SCE5_EN | 3.65E-04 | Euclidian |
| 22 | Disorder_6 score | Disorder6 | 0.00E+00 | Site |
| 23 | Weighted PSSM(C) in Voronoi neighborhood | W_PssmC_VN | 9.69E-02 | Voronoi |
| 24 | Physicochemical properties (polarity) in Euclidean neighborhood | polarity_EN | 1.07E-03 | Euclidian |
| 25 | PSSM(V) in Voronoi neighborhood | PssmV_VN | 4.57E-01 | Voronoi |
| 26 | Blosum(F) in Voronoi neighborhood | BlosumF _VN | 9.70E-04 | Voronoi |

Table S2 Benchmark dataset

| PDB | Residue ID | Residue Type | deldelG |
| --- | --- | --- | --- |
| 1DFJI | 202 | E | 1 |
| 1DFJI | 257 | W | 1.3 |
| 1DFJI | 259 | W | 2.2 |
| 1DFJI | 283 | E | 1.3 |
| 1DFJI | 285 | S | 0.8 |
| 1DFJI | 314 | W | 1 |
| 1DFJI | 316 | K | 1.3 |
| 1DFJI | 340 | E | 1.6 |
| 1DFJI | 397 | E | 1.3 |
| 1DFJI | 430 | Y | 5.9 |
| 1DFJI | 431 | D | 3.6 |
| 1DFJI | 433 | Y | 2.6 |
| 1DFJI | 453 | R | 0.8 |
| 1DFJI | 455 | I | 0.3 |
| 2PCCA | 197 | V | 2.10034 |
| 2PCCA | 290 | E | 6.19564 |
| 1JTGA | 110 | E | 4.0574 |
| 1JCKB | 23 | N | 2.5 |
| 1JCKB | 60 | N | 1.3 |
| 1JCKB | 90 | Y | 2.5 |
| 1JCKB | 91 | V | 2.16521 |
| 1JCKB | 103 | K | 0.4 |
| 1JCKB | 176 | F | 2.13155 |
| 1JCKB | 210 | Q | 2.5 |
| 1JCKA | 90 | Y | 2.546625 |
| 1DANH | 20 | K | 2.5403 |
| 1EAWA | 217 | D | 2.22665 |
| 1C08B | 32 | D | 2 |
| 1C08B | 33 | Y | 6 |
| 1C08B | 53 | Y | 3.29 |
| 1C08B | 98 | W | 5.5 |
| 1C08A | 31 | N | 5.25 |
| 1C08A | 32 | N | 5.2 |
| 1C08A | 96 | Y | 2.8 |
| 1DANT | 15 | K | -0.4 |
| 1DANT | 17 | T | 0.1 |
| 1DANT | 18 | N | 0.2 |
| 1DANT | 20 | K | 2.6 |
| 1DANT | 22 | I | 0.7 |
| 1DANT | 24 | E | 0.7 |
| 1DANT | 37 | Q | 0.55 |
| 1DANT | 41 | K | 0.35 |
| 1DANT | 42 | S | -0.1 |
| 1DANT | 43 | G | 0.2 |
| 1DANT | 44 | D | 0.7 |
| 1DANT | 45 | W | 1.6 |
| 1DANT | 46 | K | 0.25 |
| 1DANT | 47 | S | 0.05 |
| 1DANT | 48 | K | 0.4 |
| 1DANT | 51 | Y | -0.1 |
| 1DANT | 58 | D | 2.08364 |
| 1DANT | 60 | T | 2.31106 |
| 1DANT | 61 | D | 0.24 |
| 1DANT | 62 | E | 0 |
| 1DANT | 72 | L | -0.6 |
| 1DANT | 76 | F | 1.2 |
| 1DANT | 78 | Y | 0.7 |
| 1A22B | 243 | R | 2.12 |
| 1A22B | 270 | R | 0.69 |
| 1A22B | 274 | Q | 0 |
| 1A22B | 275 | E | -0.01 |
| 1A22B | 280 | W | -0.02 |
| 1A22B | 298 | S | -0.05 |
| 1A22B | 301 | T | 1.76 |
| 1A22B | 302 | S | -0.2 |
| 1A22B | 303 | I | 1.61 |
| 1A22B | 304 | W | 4.5 |
| 1A22B | 305 | I | 1.94 |
| 1A22B | 306 | P | 2.96296 |
| 1A22B | 320 | E | -0.19 |
| 1A22B | 321 | K | 0.08 |
| 1A22B | 324 | S | 0.28 |
| 1A22B | 326 | D | 0.99 |
| 1A22B | 327 | E | 0.97 |
| 1A22B | 365 | I | 2.1521 |
| 1A22B | 366 | Q | 0.02 |
| 1A22B | 367 | K | -0.02 |
| 1A22B | 369 | W | 4.5 |
| 1A22B | 371 | V | -0.64 |
| 1A22B | 417 | R | 0.28 |
| 1A22B | 419 | S | 0.03 |
| 1IARB | 13 | Y | 5.22 |
| 1IARB | 39 | L | 2.77 |
| 1IARB | 41 | F | 2.26 |
| 1IARB | 67 | D | 2.36 |
| 1IARB | 69 | V | 2.1 |
| 1IARB | 72 | D | 4.35 |
| 1IARB | 74 | Y | 2.73 |
| 1IARB | 127 | Y | 2.17 |
| 1IARB | 183 | Y | 3.68 |
| 1GC1C | 23 | S | 0.29 |
| 1GC1C | 25 | Q | 0.03 |
| 1GC1C | 27 | H | 0.28 |
| 1GC1C | 29 | K | 0.59 |
| 1GC1C | 32 | N | 0.18 |
| 1GC1C | 33 | Q | 0.1 |
| 1GC1C | 35 | K | 0.32 |
| 1GC1C | 40 | Q | -0.4 |
| 1GC1C | 42 | S | 0 |
| 1GC1C | 44 | L | 1.04 |
| 1GC1C | 45 | T | -0.15 |
| 1GC1C | 52 | N | 0.7 |
| 1GC1C | 59 | R | 1.16 |
| 1GC1C | 60 | S | -0.09 |
| 1GC1C | 63 | D | -0.32 |
| 1GC1C | 64 | Q | 0.44 |
| 1GC1C | 85 | E | 1.31 |
| 1A22A | 18 | H | -0.5 |
| 1A22A | 21 | H | 0.2 |
| 1A22A | 22 | Q | -0.2 |
| 1A22A | 25 | F | -0.4 |
| 1A22A | 26 | D | -0.2 |
| 1A22A | 42 | Y | 0.2 |
| 1A22A | 45 | L | 1.2 |
| 1A22A | 46 | Q | 0.1 |
| 1A22A | 51 | S | 0.3 |
| 1A22A | 56 | E | 0.4 |
| 1A22A | 62 | S | 0.1 |
| 1A22A | 63 | N | 0.3 |
| 1A22A | 65 | E | -0.5 |
| 1A22A | 164 | Y | 0.3 |
| 1A22A | 167 | R | 0.3 |
| 1A22A | 171 | D | 0.8 |
| 1A22A | 172 | K | 2.0064 |
| 1A22A | 175 | T | 2 |
| 1A22A | 176 | F | 1.9 |
| 1A22A | 178 | R | 2.4115 |
| 1A22A | 179 | I | 0.8 |
| 1A22A | 183 | R | 0.5 |
| 1JRHH | 32 | Y | 1.4 |
| 1JRHH | 52 | W | 2.692235 |
| 1JRHH | 53 | W | 2.40985 |
| 1JRHH | 54 | D | 2.7 |
| 1JRHH | 55 | D | 2.4 |
| 1JRHH | 56 | D | 1.9 |
| 1JRHH | 58 | Y | 1.8 |
| 1JRHH | 60 | N | 1.2 |
| 1JRHH | 100 | G | 0.54 |
| 1JRHH | 104 | G | 1.1 |
| 1JRHH | 107 | T | 1.7 |
| 1JRHL | 27 | E | 0.54 |
| 1JRHL | 30 | Y | 1.1 |
| 1JRHL | 50 | G | 4.44727 |
| 1JRHL | 91 | Y | 0.58 |
| 1JRHL | 92 | W | 2.808265 |
| 1JRHL | 93 | S | -0.65 |
| 1JRHL | 94 | T | 0.38 |
| 1JTGB | 49 | D | 2 |
| 1JTGB | 74 | K | 4.3 |
| 1JTGB | 142 | F | 2.1 |
| 1AK4C | 485 | P | 2.44716 |
| 1AK4C | 486 | V | 2.3533 |
| 1AK4C | 487 | H | 2.37151 |
| 1AK4C | 489 | G | 3.4378 |
| 1AK4C | 490 | P | 3.53324 |
| 1AK4C | 493 | P | 2.0448 |
| 2O3BB | 24 | E | 5.46881 |
| 2O3BB | 74 | Q | 3.22936 |
| 2O3BB | 76 | W | 4.06916 |
| 1DANL | 39 | L | 0 |
| 1DANL | 42 | I | 0 |
| 1DANL | 69 | I | 1.9 |
| 1DANL | 73 | L | 0 |
| 1DANL | 77 | E | 0 |
| 1DANL | 88 | Q | 0 |
| 1DANL | 92 | V | 0 |
| 1DANL | 93 | N | 0 |
| 1DANL | 94 | E | 0 |
| 1EMVB | 75 | N | 2.33298 |
| 1EMVB | 86 | F | 3.87635 |
| 3HFMY | 15 | H | -0.5 |
| 3HFMY | 20 | Y | 4.570725 |
| 3HFMY | 21 | R | 1 |
| 3HFMY | 63 | W | 0.3 |
| 3HFMY | 73 | R | -0.2 |
| 3HFMY | 75 | L | 1.25 |
| 3HFMY | 89 | T | 0 |
| 3HFMY | 96 | K | 6.9835 |
| 3HFMY | 97 | K | 5.857495 |
| 3HFMY | 98 | I | -0.1 |
| 3HFMY | 100 | S | 0.25 |
| 3HFMY | 101 | D | 11.5 |
| 1IARA | 5 | I | 1.17 |
| 1IARA | 6 | T | -0.1 |
| 1IARA | 8 | Q | -0.02 |
| 1IARA | 13 | T | -0.1 |
| 1IARA | 78 | Q | 0.13 |
| 1IARA | 81 | R | 0.48 |
| 1IARA | 84 | K | 0.35 |
| 1IARA | 85 | R | 0.43 |
| 1IARA | 88 | R | 3.75053 |
| 1H9DB | 104 | N | 2.1087 |
| 2J0TD | 2 | T | 4.38488 |
| 2J0TD | 68 | S | 2.10402 |
| 1A4YB | 5 | R | 2.3 |
| 1A4YB | 8 | H | 0.9 |
| 1A4YB | 12 | Q | 0.3 |
| 1A4YB | 13 | H | -0.3 |
| 1A4YB | 31 | R | 0.2 |
| 1A4YB | 32 | R | 0.9 |
| 1A4YB | 68 | N | 0.2 |
| 1A4YB | 84 | H | 0.2 |
| 1A4YB | 89 | W | 0.2 |
| 1A4YB | 108 | E | -0.3 |
| 1A4YB | 114 | H | 0.65 |
| 1DVFB | 32 | Y | 1.8 |
| 1DVFB | 52 | W | 4.16512 |
| 1DVFB | 54 | D | 4.2856 |
| 1DVFB | 56 | N | 1.2 |
| 1DVFB | 58 | D | 1.6 |
| 1DVFB | 98 | E | 4.189233333 |
| 1DVFB | 100 | D | 2.791766667 |
| 1DVFB | 101 | Y | 4 |
| 1NMBH | 99 | Y | 2.13919 |
| 1DVFD | 30 | K | 1 |
| 1DVFD | 33 | H | 1.9 |
| 1DVFD | 52 | D | 1.7 |
| 1DVFD | 97 | I | 2.67976 |
| 1DVFD | 98 | Y | 4.73627 |
| 1DVFD | 102 | Y | 4.7 |
| 1BRSA | 27 | K | 5.190255 |
| 1BRSA | 54 | D | -0.8 |
| 1BRSA | 58 | N | 3.091743333 |
| 1BRSA | 59 | R | 5.02655 |
| 1BRSA | 60 | E | -0.2 |
| 1BRSA | 73 | E | 2.462705 |
| 1BRSA | 87 | R | 5.667996667 |
| 1BRSA | 102 | H | 6.272162 |
| 1DVFA | 49 | Y | 1.7 |
| 1DVFA | 50 | Y | 0.7 |
| 1DVFA | 92 | W | 0.3 |
| 1KTZB | 27 | L | 2.26896 |
| 1KTZB | 30 | F | 3.42282 |
| 1KTZB | 32 | D | 1.5 |
| 1KTZB | 49 | S | 0.3 |
| 1KTZB | 50 | I | 2.34044 |
| 1KTZB | 52 | S | 0.2 |
| 1KTZB | 55 | E | 1.2 |
| 1KTZB | 77 | V | 0.4 |
| 1KTZB | 118 | D | 0.8 |
| 1KTZB | 119 | E | 1.6 |
| 1JRHI | 47 | K | 3.674256667 |
| 1JRHI | 49 | Y | 3.48318 |
| 1JRHI | 52 | K | 3.255706667 |
| 1JRHI | 53 | N | 4.16464 |
| 1JRHI | 54 | S | 0.3 |
| 1JRHI | 55 | E | -0.4 |
| 1JRHI | 82 | W | 4.450726667 |
| 1JRHI | 84 | R | -0.3 |
| 1JRHI | 98 | K | 0 |
| 1BRSD | 29 | Y | 3.43334 |
| 1BRSD | 35 | D | 4.354416667 |
| 1BRSD | 39 | D | 6.814255 |
| 1BRSD | 42 | T | 1.8 |
| 2JELP | 70 | E | 2.7253 |
| 1BXIA | 27 | T | 0.73 |
| 1BXIA | 28 | S | 0.17 |
| 1BXIA | 29 | S | 0.96 |
| 1BXIA | 33 | L | 3.42 |
| 1BXIA | 34 | V | 2.58 |
| 1BXIA | 37 | V | 1.66 |
| 1BXIA | 38 | T | 0.9 |
| 1BXIA | 41 | E | 2.08 |
| 1BXIA | 46 | H | 0.83 |
| 1BXIA | 48 | S | 0.01 |
| 1BXIA | 50 | S | 2.19 |
| 1BXIA | 51 | D | 5.92 |
| 1BXIA | 53 | I | 0.85 |
| 1BXIA | 54 | Y | 4.83 |
| 1BXIA | 55 | Y | 4.63 |
| 1KTZA | 94 | R | 2.88119 |
| 2WPTA | 37 | V | 3.80505 |
| 2WPTA | 41 | E | 4.49815 |
| 2WPTA | 50 | S | 2.423 |
| 2WPTA | 56 | P | 2.92442 |
| 1XD3B | 8 | L | 2.73662 |
| 1FFWB | 214 | F | 3.64188 |
| 1TM1I | 58 | T | 2.647415 |
| 1TM1I | 60 | E | 2.9861 |
| 1TM1I | 61 | Y | 2.711206667 |
| 1TM1I | 65 | R | 3.414125 |
| 1TM1I | 67 | R | 3.007425 |
| 1CBWD | 11 | T | 0.2 |
| 1CBWD | 15 | K | 2 |
| 1CBWD | 17 | R | 0.5 |
| 1CBWD | 19 | I | 0.1 |
| 1CBWD | 34 | V | 0 |
| 1CBWD | 39 | R | 0.2 |
| 1FCCC | 25 | T | 0.24 |
| 1FCCC | 27 | E | 4.9 |
| 1FCCC | 28 | K | 1.3 |
| 1FCCC | 31 | K | 3.48684 |
| 1FCCC | 35 | N | 2.381235 |
| 1FCCC | 40 | D | 0.3 |
| 1FCCC | 43 | W | 3.76898 |
| 1CHOI | 17 | T | 4.176475 |
| 1CHOI | 18 | L | 4.77875 |
| 1CHOI | 19 | E | 2.29936 |
| 1CHOI | 20 | Y | 2.50652 |
| 1CHOI | 21 | R | 3.14541 |
| 1FC2C | 147 | N | 0.6 |
| 1FC2C | 150 | I | 2.2 |
| 1FC2C | 154 | K | 1.2 |
| 1F47A | 5 | Y | 0.9 |
| 1F47A | 6 | L | 0.9 |
| 1F47A | 7 | D | 1.8 |
| 1F47A | 8 | I | 2.51344 |
| 1F47A | 11 | F | 2.44276 |
| 1F47A | 12 | L | 2.29277 |
| 1F47A | 14 | K | 0 |
| 1F47A | 15 | Q | 0 |
| 1DN2E | 10 | V | 2 |
| 1DN2E | 11 | W | 2 |

Table S3 Independent test dataset

| PDB | Residue ID | Label(1:hotspots,-1:non-hotspots) |
| --- | --- | --- |
| 1CDLA | 12 | -1 |
| 1CDLA | 19 | -1 |
| 1CDLA | 92 | 1 |
| 1CDLE | 799 | -1 |
| 1CDLE | 800 | 1 |
| 1CDLE | 802 | -1 |
| 1CDLE | 804 | 1 |
| 1CDLE | 808 | -1 |
| 1CDLE | 810 | 1 |
| 1CDLE | 811 | -1 |
| 1CDLE | 812 | 1 |
| 1CDLE | 813 | 1 |
| 1DVAH | 38 | -1 |
| 1DVAH | 65 | -1 |
| 1DVAH | 67 | -1 |
| 1DVAH | 70 | -1 |
| 1DVAH | 73 | -1 |
| 1DVAH | 74 | -1 |
| 1DVAH | 75 | -1 |
| 1DVAH | 76 | 1 |
| 1DVAH | 80 | -1 |
| 1DVAH | 82 | -1 |
| 1DVAH | 144 | -1 |
| 1DVAH | 153 | -1 |
| 1DVAX | 1 | -1 |
| 1DVAX | 2 | 1 |
| 1DVAX | 5 | -1 |
| 1DVAX | 7 | -1 |
| 1DVAX | 8 | -1 |
| 1DVAX | 9 | -1 |
| 1DVAX | 11 | 1 |
| 1DVAX | 12 | 1 |
| 1DVAX | 14 | -1 |
| 1DVAX | 15 | 1 |
| 1DVAX | 16 | -1 |
| 1DX5N | 24 | -1 |
| 1DX5N | 34 | -1 |
| 1DX5N | 36 | -1 |
| 1DX5N | 37 | -1 |
| 1DX5N | 38 | -1 |
| 1DX5N | 39 | -1 |
| 1DX5N | 65 | -1 |
| 1DX5N | 67 | 1 |
| 1DX5N | 74 | -1 |
| 1DX5N | 75 | -1 |
| 1DX5N | 76 | 1 |
| 1DX5N | 80 | 1 |
| 1DX5N | 81 | -1 |
| 1DX5N | 82 | -1 |
| 1DX5N | 84 | -1 |
| 1DX5N | 110 | -1 |
| 1DX5N | 235 | -1 |
| 1EBPA | 93 | 1 |
| 1EBPA | 150 | 1 |
| 1EBPA | 151 | -1 |
| 1EBPA | 205 | 1 |
| 1EBPC | 9 | -1 |
| 1EBPC | 10 | -1 |
| 1EBPC | 11 | -1 |
| 1EBPC | 12 | -1 |
| 1EBPC | 13 | 1 |
| 1ES7A | 26 | -1 |
| 1ES7A | 31 | 1 |
| 1ES7A | 49 | -1 |
| 1ES7A | 50 | -1 |
| 1FAKT | 15 | -1 |
| 1FAKT | 17 | -1 |
| 1FAKT | 18 | -1 |
| 1FAKT | 20 | 1 |
| 1FAKT | 22 | -1 |
| 1FAKT | 24 | -1 |
| 1FAKT | 37 | -1 |
| 1FAKT | 41 | -1 |
| 1FAKT | 42 | -1 |
| 1FAKT | 44 | -1 |
| 1FAKT | 47 | -1 |
| 1FAKT | 48 | -1 |
| 1FAKT | 50 | -1 |
| 1FAKT | 58 | 1 |
| 1FAKT | 94 | -1 |
| 1FAKT | 128 | -1 |
| 1FAKT | 133 | -1 |
| 1FAKT | 135 | -1 |
| 1FAKT | 140 | -1 |
| 1FAKT | 203 | -1 |
| 1FAKT | 207 | -1 |
| 1FE8A | 963 | -1 |
| 1FE8A | 987 | -1 |
| 1FE8A | 990 | -1 |
| 1FE8A | 1023 | -1 |
| 1FOEB | 41 | -1 |
| 1FOEB | 54 | 1 |
| 1G3IA | 438 | 1 |
| 1G3IA | 439 | 1 |
| 1G3IA | 441 | 1 |
| 1G3IA | 442 | 1 |
| 1G3IA | 443 | 1 |
| 1G3IA | 444 | 1 |
| 1GL4A | 403 | -1 |
| 1GL4A | 427 | 1 |
| 1GL4A | 429 | 1 |
| 1GL4A | 431 | 1 |
| 1GL4A | 440 | -1 |
| 1GL4A | 616 | 1 |
| 1GL4A | 620 | 1 |
| 1IHBB | 101 | -1 |
| 1IHBB | 133 | -1 |
| 1IHBB | 135 | -1 |
| 1IHBB | 136 | -1 |
| 1JATA | 55 | 1 |
| 1JATB | 8 | 1 |
| 1JPPB | 345 | 1 |
| 1JPPB | 354 | -1 |
| 1JPPB | 383 | 1 |
| 1JPPB | 386 | -1 |
| 1JPPB | 435 | -1 |
| 1JPPB | 469 | -1 |
| 1JPPB | 470 | -1 |
| 1MQ8B | 206 | 1 |
| 1NFIF | 181 | 1 |
| 1NFIF | 215 | -1 |
| 1NUNA | 76 | -1 |
| 1NUNA | 78 | -1 |
| 1NUNA | 155 | -1 |
| 1UB4C | 453 | -1 |
| 2HHBB | 35 | -1 |
